# Remote surveillance and detection of SARS-CoV-2 transmission among household members in King County, Washington

**DOI:** 10.1186/s12879-024-09160-z

**Published:** 2024-03-13

**Authors:** Anne Emanuels, Amanda M. Casto, Jessica Heimonen, Jessica O’Hanlon, Eric J. Chow, Constance Ogokeh, Melissa A. Rolfes, Peter D. Han, James P. Hughes, Timothy M. Uyeki, Christian Frazar, Erin Chung, Lea M. Starita, Janet A. Englund, Helen Y. Chu, Seattle Flu Study Investigators, Michael Boeckh, Michael Famulare, Barry R. Lutz, Deborah A. Nickerson, Mark J. Rieder, Matthew Thompson, Jay Shendure, Trevor Bedford, Amanda Adler, Elisabeth Brandstetter, Roy Burstein, Shari Cho, Rachel E. Geyer, James Hadfield, Michael L. Jackson, Anahita Kiavand, Ashley E. Kim, Louise E. Kimball, Jack Henry Kotnik, Kirsten Lacombe, Jennifer K. Logue, Victoria Lyon, Denise J. McCulloch, Matthew Richardson, Julia H. Rogers, Thomas R. Sibley, Monica L. Zigman Suchsland, Melissa Truong, Caitlin R. Wolf, Weizhi Zhong

**Affiliations:** 1 Division of Allergy and Infectious Diseases, Department of Medicine, University of Washington, UW Medicine Box 358061, Chu Lab Room E630, 750 Republican Street, Seattle, WA 98109 USA; 2 Influenza Division, National Center for Immunization and Respiratory Diseases, Centers for Disease Control and Prevention, Atlanta, GA USA; 3 Brotman Baty Institute for Precision Medicine, Seattle, Washington USA; 4 Department of Genome Sciences, University of Washington, Seattle, Washington USA; 5 Department of Biostatistics, University of Washington, Seattle, Washington USA; 6 Vaccine and Infectious Disease Division, Fred Hutchinson Cancer Research Center, Seattle, Washington USA; 7 Seattle Children’s Research Institute, Seattle, Washington USA; 8 Institute for Disease Modeling, Bellevue, WA USA; 9 Department of Bioengineering, University of Washington, Seattle, WA USA; 10 Bill and Melinda Gates Foundation, Seattle, WA USA; 11 Department of Family Medicine, University of Washington, Seattle, WA USA; 12 Kaiser Permanente Washington Health Research Institute, Seattle, WA USA

**Keywords:** SARS-CoV-2 infection, Remote surveillance, Household transmission, Longitudinal cohort, Whole genome sequencing

## Abstract

**Background:**

Early during the COVID-19 pandemic, it was important to better understand transmission dynamics of SARS-CoV-2, the virus that causes COVID-19. Household contacts of infected individuals are particularly at risk for infection, but delays in contact tracing, delays in testing contacts, and isolation and quarantine posed challenges to accurately capturing secondary household cases.

**Methods:**

In this study, 346 households in the Seattle region were provided with respiratory specimen collection kits and remotely monitored using web-based surveys for respiratory illness symptoms weekly between October 1, 2020, and June 20, 2021. Symptomatic participants collected respiratory specimens at symptom onset and mailed specimens to the central laboratory in Seattle. Specimens were tested for SARS-CoV-2 using RT-PCR with whole genome sequencing attempted when positive. SARS-CoV-2-infected individuals were notified, and their household contacts submitted specimens every 2 days for 14 days.

**Results:**

In total, 1371 participants collected 2029 specimens that were tested; 16 individuals (1.2%) within 6 households tested positive for SARS-CoV-2 during the study period. Full genome sequences were generated from 11 individuals within 4 households. Very little genetic variation was found among SARS-CoV-2 viruses sequenced from different individuals in the same household, supporting transmission within the household.

**Conclusions:**

This study indicates web-based surveillance of respiratory symptoms, combined with rapid and longitudinal specimen collection and remote contact tracing, provides a viable strategy to monitor households and detect household transmission of SARS-CoV-2.

**Trial registration identifier:**

NCT04141930, Date of registration 28/10/2019.

## Introduction

Since the emergence of severe acute respiratory syndrome coronavirus-2 (SARS-CoV-2), the virus that causes COVID-19, over 519 million cases have been reported worldwide, resulting in more than 6 million deaths as of May 2022 [1]. Variants of concern such as the Omicron variant have shown more effective community transmission, highlighting the urgency of mitigating the spread of this virus [2].

As transmission of SARS-CoV-2 is believed to involve inhalation of respiratory droplets or fine particulate aerosols from an infected person, household members of an infected individual may be especially at risk for infection [3–5]. Studying household SARS-CoV-2 transmission is therefore essential to understanding the transmission dynamics within the greater community. Several studies have measured the secondary incidence of SARS-CoV-2 infection in the household of an infected index case, with estimates of secondary household transmission of 16% across multiple studies, and higher in those with symptomatic index cases [6–8]. Additional methods to identify, isolate, and test household members of a SARS-CoV-2 infected individual without exposing healthcare personnel to potential infection may be beneficial both in quantifying household transmission as well as in reducing greater community spread.

Previous research has demonstrated the effectiveness of certain remote surveillance and testing methods [9–11]. However, less research has been conducted on the potential for remote contact tracing and testing of household contacts. This study of a cohort of households in the Seattle area offers data on the use of remote monitoring of SARS-CoV-2 transmission of household members of an infected person.

## Materials and methods

### Study design

From October 1, 2020, through January 31, 2021, 346 households in Washington’s (WA) Seattle metropolitan area were enrolled into a longitudinal cohort study. Enrolled households were remotely monitored weekly for symptoms of respiratory illness by an online symptom questionnaire until June 20, 2021. Households with children between 3 months and 17 years of age were recruited from local elementary and middle schools, as well as from a cohort of households participating in the prior year’s iteration of the study [12, 13]. Briefly, eligible households consisted of three or more individuals, including at least one child (aged < 18 years) and at least one adult with a computer, smart phone, or tablet that could connect to the internet, according to the design of the parent study. Interested households completed an online eligibility screening questionnaire. Eligible households and interested household members underwent informed phone consent between research staff and one adult household member. This adult household member was determined to be the ‘household reporter’. The remaining interested household members were then prompted to document their consent through the same web-based platform. Parents or guardians provided consent for children aged < 18 years, and children aged 7–17 years also provided written assent to participate in the study.

Consented household members completed an online enrollment questionnaire that collected demographic and contact information. After consent, households received four swab-collection kits per person, each containing: an anterior nasal swab, collection tube, instructions and return packaging. The household reporter then received one email or text message per week with a web link to the weekly symptom survey to be completed on behalf of all consented individuals in the household. Illness episodes in one individual prompted all participants in the household to collect an anterior nasal swab, regardless of symptoms, as soon as possible and return it to the laboratory for testing. Illness episodes were defined as new onset cough or two or more of the following respiratory symptoms: sore throat; feeling feverish; rhinorrhea; myalgia; headache; fatigue; nausea or vomiting; difficulty breathing; and rash or diarrhea (for participants < 18 years old). Symptomatic participants were asked additional questions in a follow up survey 1 week after swab collection. Households could request additional swab-collection kits if supplies were depleted.

This study was approved by the University of Washington (UW) Institutional Review Board and the Centers for Disease Control and Prevention relied on that review and approval. Enrolled households provided data in the form of questionnaires provided through Project REDCap (Research Electronic Data Capture) [14].

### Data collection

As a practice for study activities, consented participants were asked after enrollment to collect one anterior nasal swab once they received the study supplies in the mail and send it to the laboratory. Thereafter, specimens were collected from symptomatic participants and their household contacts during the study period as described below. Each anterior nasal swab was placed into a dry, 5 mL collection tube labeled with the participant’s name, the collection date, and a unique specimen identification number. The included instructions guided the participants to place their specimen tube within a sealable transport bag, and then to return the specimen bag at ambient temperature in a pre-labeled rigid container. A previous study has demonstrated the accuracy of these methods, and also determined that the laboratory-determined window for optimal specimen viability is 48 hours between swab collection and processing [15]. Initially, specimens were mailed via the United States Postal Service following International Air Transport Association (IATA) shipping procedures. Starting on December 12, 2020, specimens were transported by a courier service (Delivery Express Logistics, Seattle, WA) to ensure arrival in the laboratory within two calendar days of collection.

Beginning on October 19, 2020, unique web links to the weekly symptom logs were sent via email or text message to each designated household reporter. These logs were used to report the onset of new symptoms from any member of the household. If on the weekly symptom log any household participant experienced an acute respiratory illness (ARI; acute cough or ≥ 2 concurrent symptoms) with symptom onset within the past 72 hours, the household reporter was prompted to fill out an illness questionnaire and the individual was prompted to collect a nasal swab. Symptoms that occurred later in the week were encouraged to be reported ad hoc through the web-based survey platform using a link provided in the weekly messaging; individuals were prompted to collect a nasal swab if symptoms met the ARI criteria. Any time at least one individual in the household reported ARI, all household members was asked to collect an anterior nasal swab at the same time as the ill individual.

If SARS-CoV-2 was detected in a nasal specimen, the individual or their parent/guardian was notified by a phone call from the research team and the household was sent additional specimen collection materials for each participating member to collect a swab every other day until 14 days since the collection date of the initial SARS-CoV-2-positive swab. If additional members of the household tested positive for SARS-CoV-2, the household was notified via subsequent telephone calls from the research team but did not restart the two-week sequence.

Follow-up questionnaires were sent to any participant reporting an eligible illness episode 1 week after reporting symptoms to ask about the progression of their symptoms and any behavioral changes related to infection control precautions and their illness. If the participant tested positive for SARS-CoV-2, an additional follow up questionnaire was sent to them 2 weeks after their illness was reported. Reminders to complete questionnaires and collect swabs were delivered by automated messages and by personalized emails and phone calls from the research team.

### Laboratory testing

Specimens were transported to the Northwest Genomics Center at UW and tested for SARS-CoV-2 using a quantitative reverse transcription polymerase chain reaction (RT-qPCR) laboratory-developed test (LDT). The RT-qPCR consists of assays for 2 SARS-CoV-2 targets in duplicate and the human marker RNase P across 4 multiplexed reactions. A specimen was considered positive if 3 or 4 replicates for RNase P had a cycle threshold (Ct) value < 36 and SARS-CoV-2 had a value < 40. If only 2 SARS-CoV-2 replicate reactions were positive, the result was defined as inconclusive. For the purposes of this study, inconclusive results were regarded as positive results [16]. If SARS-CoV-2 was not detected or detected in only 1 replicate, the test was considered negative. Specimens were defined as failed and considered “never tested” if RNase P was undetected in 2 or more reactions, or if there was a laboratory or operator error. Cases were contacted by the research team as well as county or state public health staff, and contact tracing was initiated. Viral genome sequencing was attempted on all positive specimens with Ct values of ≤30 using a hybrid capture enrichment method [17] or a COVID-seq amplicon method (Illumina). Raw sequencing reads were processed into consensus genomes using the Seattle Flu Study Assembly (GitHub [18]) or a modified iVar pipeline [19]. Viral sequences were aligned and phylogenetic trees were constructed using the Nextstrain augur software [20]. Trees were visualized using the Nextstrain auspice software. All consensus genomes were publicly deposited to the Global Initiative on Sharing All Influenza Data (GISAID; gisaid.org [21]) database immediately after data generation.

## Results

From recruitment efforts, 429 households were approached among 446 households indicating interest in the study, and 346 households were enrolled. Among enrolled households, the median household size was 4 people and 145 (42%) had an annual income of over $200,000 (Table 1); median household income was approximately $95,000 [22]. Of the 1371 individual participants, 177 (13%) were children younger than 5 years of age, 358 (26%) aged 5–12 years, 106 (8%) aged 13–17 years, 595 (43%) aged 18–49 years, 119 (9%) aged 50–64 years, and 16 (1%) aged ≥65 years. Most participants (81%) were white. The first COVID-19 vaccines became available to adolescents and adults in Washington state during the course of this study, and vaccination details of this cohort will be reported elsewhere.

**Table 1:** Enrollments at a household and participant level

	**Total Participants (*n*** **= 1371)**	**No SARS-CoV-2 infection (*n*** **= 1355)**	**One or more SARS-CoV-2 infections (*n*** **= 16)**
**Age:**	**N (%)**	**N (%)**	**N (%)**
< 5 Years	177 (13)	175 (13)	2 (13)
5–12 Years	358 (26)	352 (26)	6 (38)
13–17 Years	106 (8)	106 (8)	0 (0)
18–49 Years	595 (43)	587 (43)	8 (50)
50–64 Years	119 (9)	119 (9)	0 (0)
≥ 65 Years	16 (1)	16 (1)	0 (0)
**Race**^a^** (32 missing)**			
White	1116 (81)	1101 (81)	15 (94)
Black/African American	14 (1)	13 (1)	1 (6)
Asian	93 (7)	93 (7)	0 (0)
American Indian or Alaska Native	2 (0)	2 (0)	0 (0)
Native Hawaiian or Pacific Islander	0 (0)	0 (0)	0 (0)
Two or more races selected	114 (8)	114 (8)	0 (0)
**Hispanic/Latino Ethnicity**	79 (6)	78 (6)	1 (6)
**Uses tobacco smoke or e-cigarettes**	11 (1)	11 (1)	0 (0)
**Comorbidities**			
Asthma	104 (8)	104 (8)	0 (0)
Chronic bronchitis or COPD	1 (0)	1 (0)	0 (0)
Cancer	20 (1)	20 (1)	0 (0)
Diabetes	11 (1)	11 (1)	0 (0)
Heart disease	5 (0)	5 (0)	0 (0)
Extreme obesity	3 (0)	3 (0)	0 (0)
Other condition	51 (4)	50 (4)	1 (6)
	**Total Households (*n*** **= 346)**	**No SARS-CoV-2 infection (*n*** **= 340)**	**One or more SARS-CoV-2 infections (*n*** **= 6)**
**Household size (median)**	4	5	4
**Household income**			
< $50,000	7 (2)	7 (2)	0 (0)
$50,000 - $100,000	37 (11)	37 (11)	0 (0)
$100,001 - $150,000	71 (21)	70 (21)	1 (17)
$150,001 - $200,000	55 (16)	53 (16)	2 (33)
> $200,000	145 (42)	142 (42)	3 (50)

^a^The 32 participants without race data selected ‘preferred not to say’ to this question of their Enrollment Questionnaire

A total of 2578 nasal specimens were collected by participants over the course of the study and received by the laboratory. Of the 41,324 symptom logs provided by participating households (92% of expected responses), 395 (0.96%) indicated a household member was experiencing an ARI episode and prompted swab collection; 371 (94%) of these symptomatic participants sent a corresponding nasal swab to the laboratory. An additional 1056 nasal specimens were collected from household contacts of symptomatic participants experiencing an ARI episode (98% of expected responses). Of the specimens received by the lab, a total of 2029 specimens (79%) were fully processed. A majority of the specimens (87%) that were unable to be processed arrived in the laboratory shortly after the two-calendar-day window of time in which the specimen was considered approved for testing. Most failed specimens were shipped before December 12, 2020, when the shipping procedures were modified to use a local logistics company (see Methods).

**Fig. 1:**
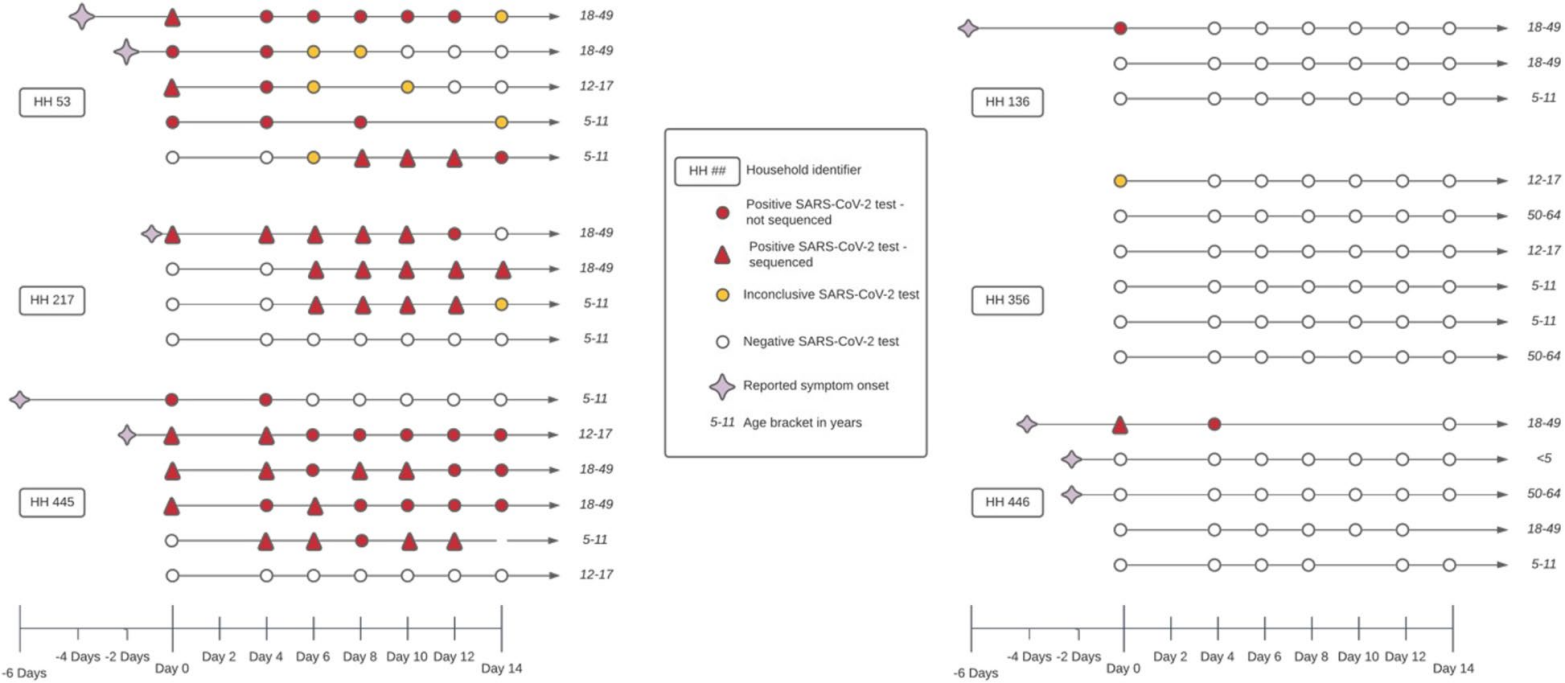
Depiction of the follow-up period and test results for 6 households where at least 1 individual had a positive SARS-CoV-2 test. Clusters of lines indicate households, with each line representing a single individual. Circles represent a nasal specimen was taken and the circle is colored red if the specimen was positive for SARS-CoV-2, yellow if the test was inconclusive, and white if the test was negative. Triangles indicate that the positive specimen was eligible for whole genome sequencing

A total of 16 individuals among 6 households tested positive or inconclusive for SARS-CoV-2 during the study (Fig. 1). The 14-day follow-up period began 4 days after the initial positive swab and 164 nasal specimens were collected from household members (94% of expected). Based on self-reported date of symptom onset, a child in the household between the ages of 12–17 years appeared to be the index case in one household, while an adult experienced either the initial onset of symptoms or the first positive test result in the remaining five households. In 3 (50%) of these households, transmission of SARS-CoV-2 between household contacts was observed. In only one household (HH 53) did all participants test positive at least once during the 14-day follow-up.

**Fig. 2:**
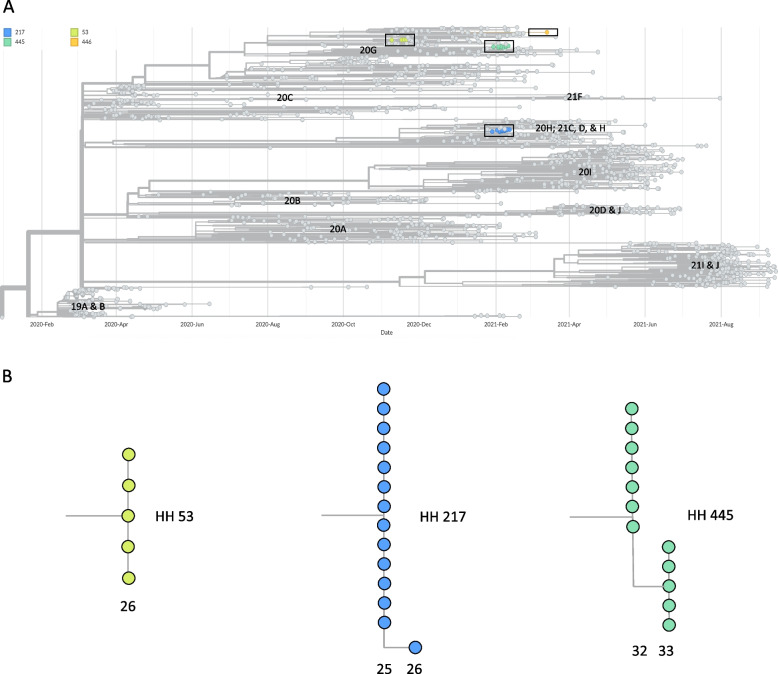
Phylogeny of SARS-CoV-2 samples from households. **A** Phylogenetic tree containing all 32 genomes generated from SARS-CoV-2 samples collected as part of the household study. These genomes are labeled by household by the colors shown in the legend and boxes enclose genomes from the same household. Gray nodes in the tree represent SARS-CoV-2 genomes from samples collected in Washington in 2020 and 2021 and deposited in the GISAID database. The x-axis of the tree represents collection date. Sequence clusters are labeled according to nextstrain clade. **B** Detailed view of branches containing genomes from households 53, 217, and 445. The x-axis of the branches corresponds to number of nucleotide changes the genomes are from the SARS-CoV-2 reference sequence given by the numbers below the branches

Full genome sequences were generated for 32 SARS-CoV-2 positive specimens from 11 unique individuals (Table 2); these individuals resided in 4 different households including all 3 households (HH 53, HH 217, and HH 445) where suspected transmission among household members had occurred (Fig. 1). More than one positive specimen was sequenced for some individuals. Genome sequences for the remaining positive and inconclusive specimens could not be generated due to insufficient viral ribonucleic acid quality and/or quantity. Figure 2 shows a phylogenetic tree that includes the genomes for 32 specimens from this study, the Wuhan/Hu-1 reference genome, and 1570 publicly available SARS-CoV-2 genomes generated from specimens collected in King County, WA from February 2020 to September 2021.

**Table 2:** Number of specimens with full SARS-CoV-2 genomes from households and individuals

Household	# of Genomes	NextStrain Clade	Pangolin Lineage	Variants Unique to Household^a^	Individual	# of Genomes	Genotypes at Positions Variable within Households^b^
53	5	20G	B.1.2	G5273A	53.1	1	NA
					53.3	1	NA
					53.5	3	NA
217	14	21C	B.1.429	A10761G	217.1	5	22018 T
					217.2	5	22018C (Specimen 2) 22018 T (Specimen 1, 3, 4) Missing Data (Specimen 5)
					217.3	4	22018 T
445	12	20G	B.1.2	T19389G	445.1	4	12789 T
					445.2	2	12789C
					445.4	2	12789C (Specimen 1) 12789 T (Specimen 2)
					445.6	4	12789C
446	1	20G	B.1.2	A5476C, G20383A, G24586T	446.7	1	NA

^a^Relative to publicly available SARS-CoV-2 genomes from King County, WA deposited in GISAID

^b^NA (not applicable) when all of the genomes for viruses from a household were found to be identical

All sequenced specimens from 3 households (53, 445, 446) were found to be the NextStrain Clade 20G/Pangolin Lineage B.1.2. (Table 2). All sequenced specimens from the remaining household (217) were NextStrain Clade 21C/Pangolin Lineage B.1.429/Epsilon variant. All consensus genomes from HH 53, HH 217, and HH 445 share variant alleles 5273A, 10761G, and 19389G, respectively, which distinguish genomes from these households from publicly available genomes from King County deposited in GISAID. There was also high bootstrap support (100% for each household group) for all sequences from HH 53, HH 217, and HH 445, respectively, forming monophyletic groups exclusive of other sequences from King County. All consensus genomes from HH 53 are identical while genomes from HH 217 and HH 445 vary at position 22018 and 12789, respectively.

The approximate cost estimate for a contact tracing event for a household of four individuals is $213.38 (personal communication, Zack Acker). This is based on 2 weeks of testing for a 4-person household, with an estimated four swabs per person for a total of 16 swabs. We estimate that 2 weeks of household surveillance costs $58.91, with labor costs for fabrication and sending and receiving kits at $35.19. The transportation cost to use a courier service is estimated at $24 per trip, with one outbound trip and four return trips with batched samples for a total of five trips and a total transport cost of $120.

## Discussion

In this study, we demonstrated the successful implementation of remote SARS-CoV-2 surveillance and contact tracing in a cohort of households in the Seattle metropolitan area. Participants were successfully monitored for episodes of symptomatic illness over the course of the study, and cases of SARS-CoV-2 were detected in time to initiate 2 weeks for follow up specimen collection procedures. These methods indicate a practical and innovative approach to remote assessment of SARS-CoV-2 and other respiratory virus burden among community households while minimizing health care utilization and exposure risk.

There have been several studies to measure the transmission of SARS-CoV-2 between household members, including prospective, longitudinal cohorts that self-collected nasal swabs weekly [23]. Most, however have initiated follow-up of household members once a positive individual was detected, which typically has occurred at a lag of 4–10 days after the symptom onset in the initial index case [8, 6, 24–27]. The model used in this study, pre-positioned specimen collection kits within households such that every member of the household could provide a specimen as soon as the initial illness in the household was reported. This model shortened the time to the first swab in a household member and presents a cohesive strategy to quickly detect SARS-CoV-2 transmission events that does not require the resources and cost of weekly specimen collection. In this study, we estimated that the total cost for remote contact tracing is approximately 200 USD. We acknowledge that the costs of web-based surveillance and remote contact tracing may appear high; however, there is also a cost associated with contact tracing done through in-person surveillance by case workers, as is often done for other communicable diseases such as tuberculosis.

In the three households where additional SARS-CoV-2 infections were detected, the timing of the sequential SARS-CoV-2 detections supported the likelihood of household transmission. Additionally, very little genetic variation at the consensus level was found among viral specimens from different individuals in the same household (maximum pairwise genetic distance of one single nucleotide change), consistent with intra-household viral spread. Sequencing in this study allowed for assessment of within household transmission of the same strain and may be most useful in a setting where multiple different variants are co-circulating; however, in a resource-constrained setting, sequencing could be eliminated or used only on the first samples from individuals.

There were several limitations to this study. First, while the study identified symptomatic index cases of COVID-19, it did not allow for detection of asymptomatic or minimally symptomatic SARS-CoV-2 introduction to the household. As mild and asymptomatic cases of SARS-CoV-2 are common, especially among vaccinated individuals, this was a limitation that should be addressed in future iterations of this testing model [28]. Second, there were a notable number of specimens that were unable to be tested because of packaging errors, shipping errors, or delays in arrival to the laboratory. While these errors diminished in frequency over the course of the study with improvements in shipping techniques, improvements in packaging and instructions could further reduce these errors. Importantly, the study population was not reflective of the Seattle area nor the general United States and therefore our conclusions may not be generalizable to the wider community. Importantly, our study method depends upon reliable transport of specimens from homes to laboratories and shipping within this study in the Seattle area may not reflect the access and timeliness of shipping or transport in other parts of the United States or globally. However, we believe that the use of remote contact tracing methods can be applied to global populations, particularly in places where there are health care worker shortages, shifting the collection of data and samples to the household itself with use of text messaging, self-collection of nasal swabs, and return of kits to the clinics has the possibility to reduce the overall burden on the health care system. Other advantages of remote contact tracing may include faster epidemic control than traditional methods, as has been shown in some modeling studies [29, 30].

## Conclusions

In conclusion, web-based surveillance of respiratory symptoms combined with specimen collection and remote contact tracing provides a strategy to monitor households for the onset of SARS-CoV-2 infection and quickly capture transmission events. As communities in the United States seek methods to monitor for infection and respond quickly to transmission events, this strategy may offer a convenient and reliable method in households with children.

## Data Availability

The datasets used and/or analyzed during the current study are available from the corresponding author on reasonable request. All consensus genomes were publicly deposited to the Global Initiative on Sharing All Influenza Data (GISAID; gisaid.org [21]) database immediately after data generation. The GISAID accession numbers for SARS-CoV-2 positive samples utilized in this analysis are listed here:

**Table:** 

GISAID name	GISAID Accession
**USA/WA-S11469/2020**	EPI_ISL_4636179
**USA/WA-S11470/2020**	EPI_ISL_4636180
**USA/WA-S11472/2020**	EPI_ISL_4636182
**USA/WA-S11473/2020**	EPI_ISL_4636183
**USA/WA-S11474/2020**	EPI_ISL_4636184
**USA/WA-S11502/2021**	EPI_ISL_4636212
**USA/WA-S11503/2021**	EPI_ISL_4636213
**USA/WA-S11504/2021**	EPI_ISL_4636214
**USA/WA-S11505/2021**	EPI_ISL_4636215
**USA/WA-S11507/2021**	EPI_ISL_4636217
**USA/WA-S11508/2021**	EPI_ISL_4636218
**USA/WA-S11509/2021**	EPI_ISL_4636219
**USA/WA-S11510/2021**	EPI_ISL_4636220
**USA/WA-S11511/2021**	EPI_ISL_4636221
**USA/WA-S11512/2021**	EPI_ISL_4636222
**USA/WA-S11513/2021**	EPI_ISL_4636223
**USA/WA-S11514/2021**	EPI_ISL_4636224
**USA/WA-S11515/2021**	EPI_ISL_4636225
**USA/WA-S11516/2021**	EPI_ISL_4636226
**USA/WA-S11517/2021**	EPI_ISL_4636227
**USA/WA-S11518/2021**	EPI_ISL_4636228
**USA/WA-S11519/2021**	EPI_ISL_4636229
**USA/WA-S11520/2021**	EPI_ISL_4636230
**USA/WA-S11521/2021**	EPI_ISL_4636231
**USA/WA-S11495/2021**	EPI_ISL_4636205
**USA/WA-S11496/2021**	EPI_ISL_4636206
**USA/WA-S11499/2021**	EPI_ISL_4636209
**USA/WA-S11501/2021**	EPI_ISL_4636211
**USA/WA-S11524/2021**	EPI_ISL_4636234
**USA/WA-S11522/2021**	EPI_ISL_4636232
**USA/WA-S11493/2021**	EPI_ISL_4636203
**USA/WA-S11494/2021**	EPI_ISL_4636204

## Abbreviations

ARI: Acute respiratory illness
Ct: Cycle threshold
GISAID: Global initiative on sharing all influenza data
LDT: Laboratory-developed test
IATA: International air transport association
REDCap: Research electronic data capture
RT-qPCR: Reverse transcription polymerase chain reaction
SARS-CoV-2: Severe acute respiratory syndrome coronavirus-2
SIR: Secondary incidence rate
UW: University of Washington
WA: Washington
